# Characterization of the Myeloid Cell Populations’ Resident in the Porcine Palatine Tonsil

**DOI:** 10.3389/fimmu.2018.01800

**Published:** 2018-08-15

**Authors:** Ferran Soldevila, Jane C. Edwards, Simon P. Graham, Lisa M. Stevens, Bentley Crudgington, Helen R. Crooke, Dirk Werling, Falko Steinbach

**Affiliations:** ^1^Virology Department, Animal and Plant Health Agency, Addlestone, United Kingdom; ^2^The Pirbright Institute, Pirbright, United Kingdom; ^3^School of Veterinary Medicine, University of Surrey, Guildford, United Kingdom; ^4^Department of Pathobiology and Population Sciences, The Royal Veterinary College, Hatfield, United Kingdom

**Keywords:** dendritic cells, macrophages, myeloid, porcine, palatine tonsil, immunology, pig

## Abstract

The palatine tonsil is the portal of entry for food and air and is continuously subjected to environmental challenges, including pathogens, which use the tonsil and pharynx as a primary site of replication. In pigs, this includes the viruses causing porcine respiratory and reproductive syndrome, and classical and African swine fever; diseases that have impacted the pig production industry globally. Despite the importance of tonsils in host defense, little is known regarding the phenotype of the myeloid cells resident in the porcine tonsil. Here, we have characterized five myeloid cell populations that align to orthologous populations defined in other mammalian species: a CD4^+^ plasmacytoid dendritic cell (DC) defined by expression of the conserved markers E2.2 and IRF-7, a conventional dendritic cell (cDC1) population expressing CADM1^high^CD172a^low^ and high levels of XCR1 able to activate allogeneic CD4 and CD8 T cells; a cDC2 population of CADM1^dim^ cells expressing FLT3, IRF4, and CSF1R with an ability to activate allogeneic CD4 T cells; CD163^+^ macrophages (Mϴs) defined by high levels of endocytosis and responsiveness to LPS and finally a CD14^+^ population likely derived from the myelomonocytic lineage, which showed the highest levels of endocytosis, a capacity for activation of CD4^+^ memory T cells, combined with lower relative expression of FLT3. Increased knowledge regarding the phenotypic and functional properties of myeloid cells resident in porcine tonsil will enable these cells to be targeted for future vaccination strategies to current and emerging porcine viruses.

## Introduction

Pigs are both an important source of meat globally and represent a valuable biomedical model. The porcine and human immune systems present evolutionary convergent features and, as such, pigs represent an important model for disease pathogenesis and vaccine development (1). In pigs, however, the mononuclear phagocyte system composed by dendritic cells (DCs), monocytes and macrophages (Mϴs) is less characterized than those of either mice or humans. As a first line of defense to pathogen invasion, a clearer understanding of these cells, and how they might be identified, will facilitate our understanding of host–pathogen interaction in this species.

Dendritic cells are the sentinels of the immune system, they possess a distinct morphology and a unique capacity to activate naïve T cell populations (2, 3). They are also able to coordinate or regulate the adaptive immune system, depending on the antigenic signals and microbial environment at the time of antigen uptake. DCs are classified into two populations; plasmocytoid dendritic cells (pDCs), specializing in the production of type I IFNs and conventional DC (cDCs), which are potent antigen-presenting cells (4, 5). Two subpopulations of cDCs (cDC1s and cDC2s) have been described in mouse and human (6) and more recently in other mammalian species (7–11). Across species, these populations share expression of several conserved phenotypic markers, cytokine secretion profiles, and specific functionalities. However, while cDC1s are presumed unique in their capacity to cross-present antigen to CD8 T cells in mice (12), both cDC1 and cDC2 appear able to cross present in humans, depending on specific Toll-like receptor (TLR) stimulation and the local cytokine environment (13, 14) indicating that these subsets may have some redundant functions. During an immune response, an additional “inflammatory” DC subset has been identified (moDC) in lymphoid tissue, which in mice are recruited from circulating Ly6C^high^ monocytes (15). This population is capable of presenting antigen to both CD4 and CD8 T cells and inducing T_H_1, T_H_2 (15, 16), or T_H_17 mediated responses (17, 18).

Mϴs are also resident in lymphoid (and non-lymphoid) tissues that have developed from either early erythro-myeloid progenitors from the extra-embryonic yolk sac or which have matured from circulating monocytes (19). These cells are characterized by their variable expression of CD14, their responses to TLR4 stimulation, active phagocytic properties, and their production of inflammatory cytokines such as TNF-α, IL1β, IL-6, IL-8, and IL-12 (20).

In pigs, DCs and Mϴ/monocyte populations have been characterized successfully in skin (7, 8), blood (21–23), lungs (24), and lymphoid tissue (25). In the skin, CD172a^neg^CD163^neg^ cells were identified as cDC1 cells given their high expression of CADM1 and XCR1. The CD163^low^CD172a^pos^ cells expressed markers ZBTB46 and FLT3 aligning them with mouse and human cDC2s. A population of CD163^pos^ porcine dermal dendritic cell were also classified as similar to human CD14^+^ dermal DCs (7, 8). Applying a similar panel of antibodies, cDC1 and cDC2 populations were identified in porcine lungs (24).

The palatine tonsil is positioned at the opening of the respiratory and gastrointestinal tract, providing an immunological barrier (consisting of Mϴs, DCs, and lymphocytes) equipped to induce an immune response. In human tonsils, three populations of DCs have been described; pDCs, cDC1s, and cDC2s (26) and more lately, cDC1s and cDC2s have been identified in the porcine tonsil (25). However, the different populations that make up the milieu of myeloid cells, which reside in the porcine tonsil has received little attention. It is these cells, which form the first line of defense to air-borne pathogens and viruses and are tasked with ensuring an appropriate immune response is relayed following encounter with pathogenic or commensal-derived microbes. Here, we successfully employed multi-parameter flow cytometry to immunophenotype five distinct myeloid cell populations resident in porcine tonsil. To further characterize these populations, we localized these subsets *in situ* using confocal microscopy, sorted and assessed these cells functionally and, by way of quantitative RT-PCR (RT-qPCR), evaluated the expression of conserved markers expressed by various myeloid cells populations. Through these analyses, we identified three orthologous classical DC subsets (pDCs, cDC1s, and cDC2s), Mϴs, and a CD14-positive subset with characteristics interrelating with DCs and Mϴs, consistent with a monocyte-derived DC population.

## Materials and Methods

### Animals and Tissue Collection

Pig palatine tonsils were obtained from a local abattoir and transported at room temperature to the laboratory. Pigs were typically 6- to 12-month-old Large White or Large White crossbreeds. For the mixed leukocyte reaction (MLR), peripheral blood mononuclear cells (PBMC) were isolated from blood obtained from animals kept at the Animal and Plant Health Agency (APHA) facilities under housing and sampling regulations approved by the APHA Animal Welfare and Ethical Review Board and conducted in accordance with the Animals (Scientific Procedures) Act, UK.

### Tonsil Cell Isolation and Lymphocyte Depletion

Porcine palatine tonsils were dissected from the surrounding tissue and washed twice with PBS before being placed in a Petri dish. Tonsils were then cut into small fragments while submerged in PBS and further dissociated using the perforated end of a syringe plunger. The resulting cell suspension was filtered through a 40 µm cell strainer (Corning, Sigma-Aldrich, Gillingham, UK) and mononuclear cells were then separated over a Ficoll gradient (1.077 g/l, Sigma-Aldrich). Myeloid cells were enriched by magnetic depletion of lymphocytes using anti-CD3 (clone 8E6), anti-CD8α (clone PT36A) (both from Washington State University Monoclonal Antibody Center, Pullman, WA, USA), anti-CD21 (clone BB6-11C9.6, Cambridge Bioscience, Cambridge, UK), and anti-IgM (Clone K52 1C3; Bio-Rad AbD Serotec Ltd., Oxford, UK) mAbs followed by incubation with anti-mouse IgG1 magnetic beads and separation through LD columns (Miltenyi Biotech, Bisley, UK) according to the manufacturer’s instructions.

### Flow Cytometry and Cell Sorting

For phenotypic analysis of tonsillar myeloid cells, cell surface staining was performed in three consecutive steps. Cells were initially incubated with the same lymphocyte lineage antibodies as described above (anti-CD3, anti-CD8α, anti-CD21, and anti-IgM, all of an IgG1 isotype) and anti-CD4-PerCP-Cy5.5 (clone 72-12-4; BD Pharmingen, Oxford, UK), CD14 PE Texas Red (clone Tük4; Fisher Scientific, Loughborough, UK), MHC class II-DR (clone 2E9/13; Bio-Rad AbD Serotec Ltd.) labeled with Zenon anti-mouse IgG2b PE (Life Technologies, Paisley, UK), and anti-Syn-CAM (TSLC1/CADM1) biotinylated antibody (Clone 3E1; MBL, Caltag Medsystems, Buckingham UK). Following incubation for 10 min at room temperature (rt), cells were washed and then labeled with a secondary anti-mouse IgG1 Brilliant Violet 421 (Clone RMG1-1; BioLegend, London, UK) and streptavidin Brilliant Violet 605 (BioLegend) again for 10 min at rt. Finally, cells were stained with anti-CD172a FITC (clone BL1H7; Bio-Rad AbD Serotec Ltd.) and anti-CD163 conjugated to Zenon anti-mouse IgG1 APC (Life Technologies), again for 10 min at rt. For staining of CD80/86, CD163 was conjugated to Zenon anti-mouse IgG1 APC Alexa-fluor 750 and CD152 (CTLA-4)-mIg, which binds to CD80/86, (Ancell, Bayport, MN, USA) was conjugated to Zenon anti-mouse IgG2a APC. Data were acquired on a LSRII Fortessa (BD Biosciences, Oxford, UK) and collected in FACS Diva Software (BD Biosciences). All analysis and compensation was performed using Kaluza Software (Beckman Coulter, High Wycombe, UK).

For several downstream analyses, the identified myeloid populations were stained as described above and sorted using a MoFLo Astrios (Beckman Coulter). Sorted populations were collected in RPMI-1640 medium supplemented with 40% fetal bovine serum and 100 U/mL of penicillin, 100 μ/mL streptomycin (Life Technologies). For mRNA extraction, cells were centrifuged and supernatant removed before snap freezing in liquid nitrogen. Cells were stored at −80°C until RNA extraction. Typically, between 3 and 8 × 10^5^ cells were analyzed by flow cytometry (per sample) depending on the experiment. For sorting, between 5 and 10 × 10^6^ cells were sorted depending on the pig.

### TLR Stimulation and Intracellular Cytokine Staining

Lymphocyte-depleted tonsillar mononuclear cells from eight pigs (obtained from an abattoir) and isolated as described above and dispensed in to round-bottom 96-well plates in 200 µl of complete RPMI-1640 (cRPMI), supplemented with 10% FBS, 100 U/ml penicillin, and 100 µg/ml streptomycin (Life Technologies). Cells were cultured for 10 h with either CpG ODN21798 (to stimulate TLR9) (Miltenyi Biotec, Bisley UK) at 10 µg/ml, Poly I:C HMW (to stimulate TLR3) (Invivogen, Toulouse, France) at 10 µg/ml, LPS (to stimulate TLR4) (Invivogen) at 1 µg/ml or media supplemented with recombinant IL-3 at 10 ng/ml at 37°C + 5% CO_2_. Following 4 h of culture, GolgiPlug^®^ (BD Biosciences) was added and cells were incubated for a further 6 h. Cells were then stained as described above with the exception of applying non-biotinylated anti-Syn-CAM (TSLC1/CADM1) (Clone 3E1; MBL) (Caltag Medsystem) labeled with anti-chicken IgY APC (Jackson ImmunoResearch, Newmarket, UK) and CD163 conjugated with Zenon anti-mouse IgG1 APC AlexaFluor750 (Life Technologies, Paisley, UK). For intracellular staining, cells were treated with BD Cytofix/Cytoperm™ (BD Biosciences, Oxford, UK) for 20 min at 4°C washed with BD Perm/Wash™ before staining with either biotinylated anti-IL-12 (R&D Systems, Abingdon, UK) or directly conjugated anti-TNF-α Brilliant Violet 605 (eBioscience, Hatfield, UK) in Perm/Wash™ buffer. IL-12 staining was detected by addition of streptavidin BV605 for 30 min at 4°C. Finally, cells were washed with Perm/Wash™ and resuspended in PBS supplemented with 2% FBS. Staining was assessed on the LSRII Fortessa.

### OVA Processing by Tonsillar Myeloid Cells

Lymphocyte-depleted tonsillar mononuclear cells from four pigs were applied at 2.5 × 10^5^ cells/well in cRPMI-1640. DQ-OVA FITC (Life technologies) at a final concentration of 2 µg/ml, or media (as a negative control), were added in triplicate to the cells and cultured at either 37 or 4°C (to confirm active uptake of antigen). After 1.5 h incubation, cells were stained using the phenotypic staining protocol described above with the exception that CD172a-FITC was substituted with CD172a (non-conjugated) labeled with Zenon anti-mouse IgG1 APC (Life Technologies). Samples were acquired on the LSRII Fortessa.

### Mixed Lymphocyte Reaction

For allogeneic T cell stimulation experiments, PBMCs were stained using CellTrace™ Violet Cell Proliferation Kit (Life Technologies). 10^6^ cells were incubated with 5 µM of dye in PBS (at 37°C for 20 min). Cells were washed and resuspended in cRPMI-1640 and incubated for a further 10 min. Sorted myeloid cells were cultured at 5 × 10^3^ cells/well, and a total of 2.5 × 10^4^ PBMC were added to obtain a 1:5 ratio (APC:T cell) in a final volume of 200 µl in either duplicate or triplicate depending on the number of myeloid cells successfully sorted. Negative and positive controls were included using CellTrace™ Violet stained PBMCs with media and pokeweed mitogen (PWM; Sigma-Aldrich) at a concentration of 10 µg/ml, respectively. After 5-day culture, cells were washed and stained with anti-CD4-PerCP-Cy5.5 (clone 74-12-4; BD Pharmingen) anti-CD8a-PE (clone 76-2-11; BD Pharmingen) and analyzed by flow cytometry. Reduction of CellTrace™ Violet staining was evaluated as an indicator of cell proliferation, whereby CD4^+^CD8a^−^ (naïve CD4^+^ T helper cells), CD4^−^CD8α^+^ (cytotoxic T lymphocytes), and CD4^+^CD8α^+^ (CD4^+^ T memory cells) were identified and the percentage of proliferating cells in each population determined. To identify the relative proliferation index of each of the T cell populations, a value of 100 was assigned to the myeloid cell population stimulating the maximum percentage of proliferating cells and other populations were normalized to this.

### RNA Extraction and RT-qPCR

Total RNA from FACSorted cell populations was extracted using the RNeasy Micro Kit (QIAGEN, Manchester, UK) according to the manufacturer’s instructions. Genomic DNA was removed using the RNase-Free DNase step (Qiagen) during RNA extraction. RNA was reverse transcribed using random hexamers and the M-MLV reverse transcriptase (Promega, Southampton, UK). All qPCR reactions were performed using SYBR^®^ Select Master Mix (ThermoFisher Scientific, Paisley, UK) in a final volume of 20 µl. The primers used are listed in Table 1. Analysis was performed using the MxPro QPCR Software (Agilent Technologies, Stockport, UK) and the cycle threshold (*C*_T_) values for each amplification curve were determined. Relative quantification was calculated using the Δ*C*_T_ method and normalized to the expression of β-actin mRNA. In order to compare data sets, for each gene, the cell populations with the highest level of expression was considered 100, and the remaining populations were expressed as a percentage of that value as shown by Maisonnasse and colleagues (24).

**Table 1:** Primers used for qPCR.

Target mRNA	Primer sequence	Reference
CSF1R	Fwd: 5′-TGAACGACTCCAACTACATTGTCA-3′ Rev: 5′-TGTAGACGCAGTCGAAGATGCT-3′	(8)
E2.2	Fwd: 5′-CCTTCTCTCTCAGCAGGCAC-3′ Rev: 5′-CAGACGACCCTTTGCTCCAT-3′	Designed
IRF7	Fwd: 5′-TGGCAGCACATACTGGTGAG-3′ Rev: 5′-AGTGGGCCTGCATATGGAAC-3′	Designed
XCR1	Fwd: 5′-CGATGCCGTCTTCCACAAG-3′ Rev: 5′-GGAACCACTGGCGTTCTGA-3′	(8)
IL-1b	Fwd: 5′-AGAGATGAAGTGCTGCACCC-3′ Rev: 5′-ACAGACAAAGTCATCATTGCACG-3′	Designed
IRF4	Fwd: 5′-CCGGCCTGTGAAAATGGTTG-3′ Rev: 5′-GGACGTGGTCAGCTCTTTCA-3′	Designed
ZBTB46	Fwd: 5′-GCTGGTGCACAGCAAGGA-3′ Rev: 5′-GCGGCCGACATGAACAC-3′	(8)
MAFB	Fwd: 5′-TGCGTTCTTTAGACCAATATGTTATGT-3′ Rev: 5′-CACCAATAACTCGCCCGCTAT-3′	(8)
FLT3	Fwd: 5′-TGTTCACGCTGAATATAAGAAGGAA-3′ Rev: 5′-GGAGCAGGAAGCCTGACTTG-3′	(8)
SIRPα	Fwd: 5′-CTGAGACCATCCGAGTTCCG-3′ Rev: 5′-CACGCCCACCGTGATAAAGA-3′	Designed
β-actin	Fwd: 5′-GACTCAGATCATGTTCGAGACCTT-3′ Rev: 5′-CATGACAATGCCAGTGGTGC-3′	Designed
TLR1	Fwd: 5′-AGATTTCGTGCCACCCTATG-3′ Rev: 5′-CCTGGGGGATAAACAATGTG-3′	(27)
TLR2	Fwd: 5′-TGCTATGACGCTTTCGTGTC-3′ Rev: 5′-CGATGGAGTCGATGATGTTG-3′	(27)
TLR3	Fwd: 5′-GAGCAGGAGTTTGCCTTGTC-3′ Rev: 5′-GGAGGTCATCGGGTATTTGA-3′	(27)
TLR4	Fwd: 5′-TCATCCAGGAAGGTTTCCAC-3′ Rev: 5′-TGTCCTCCCACTCCAGGTAG-3′	(27)
TLR5	Fwd: 5′-GGTCCCTGCCTCAGTATCAA-3′ Rev: 5′-TGTTGAGAAACCAGCTGACG-3′	(27)
TLR6	Fwd: 5′-TCAAGCATTTGGACCTCTCA-3′ Rev: 5′-TTCCAAATCCAGAAGGATGC-3′	(27)
TLR7	Fwd: 5′-TCTGCCCTGTGATGTCAGTC-3′ Rev: 5′-GCTGGTTTCCATCCAGGTAA-3′	(27)
TLR8	Fwd: 5′-CTGGGATGCTTGGTTCATCT-3′ Rev: 5′-CATGAGGTTGTCGATGATGG-3′	(27)
TLR9	Fwd: 5′-GGCCTTCAGCTTCACCTTGG-3′ Rev: 5′-GGTCAGCGGCACAAACTGAG-3′	(21)
TLR10	Fwd: 5′-GCCCAAGGATAGGCGTAAAT-3′ Rev: 5′-CTCGAGACCCTTCATTCAGC-3′	(27)

*IL, interleukin; qPCR, quantitative PCR; TLR, toll-like receptor*.

### Confocal Microscopy

Optimum cutting temperature media (OCT) (Sakura Finetek UK Ltd, UK) treated tissue blocks were submerged in isopentane at −80°C until frozen and cut into 6–10 µm thick sections by cryo-sectioning (Leica RM2135 cryotome). Sections were then transferred onto microscopy slides and fixed in absolute ethanol before storing at −80°C prior to processing.

The mounted tissue sections were placed into Sequenza clips (Shandon, Paisley, UK) and then incubated in 5% (w/v) normal goat serum (Sigma-Aldrich) in TBS-T either overnight (panel 1) or for 30 min (panel 2) at room temperature in the sequenza staining rack. For identification of CD14^+^ cells and pDCs, panel 1 antibodies were applied, these included lineage antibodies (as described above; conjugated to biotin), Anti-CD4α Alexa Fluor 488 (AF488) (Clone MIL17; Bio-Rad), and Anti-CD14 DyLight 550 (Clone Tük4), all antibodies were conjugated to their respective fluorochrome using Innova Lightning-Link™ Labeling Kits (Expedeon). Panel 1 antibodies were applied to the slides for 120 min at 37°C, then washed three times in TBST before incubation with streptavidin-APC (Bio-Rad) at 37°C for a further 90 min before washing again for three times in TBST. The slides were then incubated with 4’,6-Diamidino-2-Phenylindole, Dihydrochloride (DAPI, Thermo Fisher) diluted 1/10,000 in deionized water at room temperature for 30 min before washing twice. For detection of cDC1s, cDC2s, and Mϴs, panel 2 antibodies were applied for 30 min at room temperature, which included the lineage antibodies (unconjugated). After washing three times with TBST, anti-mouse IgG1-Brilliant Violet 421 (BV421) (Clone RMG1-1; BioLegend) was applied for detection. After further washing steps, anti-CD172a FITC (clone BL1H7), anti-Syn-CAM (TSLC1/CADM1) biotinylated antibody (Clone 3E1), and anti-CD163 (clone 2A10/11; Bio-Rad) conjugated to anti-mouse IgG1 Zenon APC (Life Technologies) were added for 30 min at room temperature. Slides were then washed before applying streptavidin Brilliant Violet 605 (BioLegend) for 30 min at room temperature.

Finally, all the slides were washed twice with deionized water before being removed from the Sequenza clips and coverslips mounted with Pro-Long Gold anti-fade mounting media (Thermo Fisher). The slides were allowed to dry in the dark overnight and then sealed with nail varnish. Slides were imaged using the Leica SP2 confocal microscope.

### Statistical Analysis

GraphPad Prism 6.0 (GraphPad software, La Jolla, CA, USA) was used for the analysis of data sets. Statistical tests applied to each dataset are indicated in the relevant figure legend.

## Results

### Identification of Five Distinct Populations of Myeloid Cells in Porcine Tonsil

**Figure 1:**
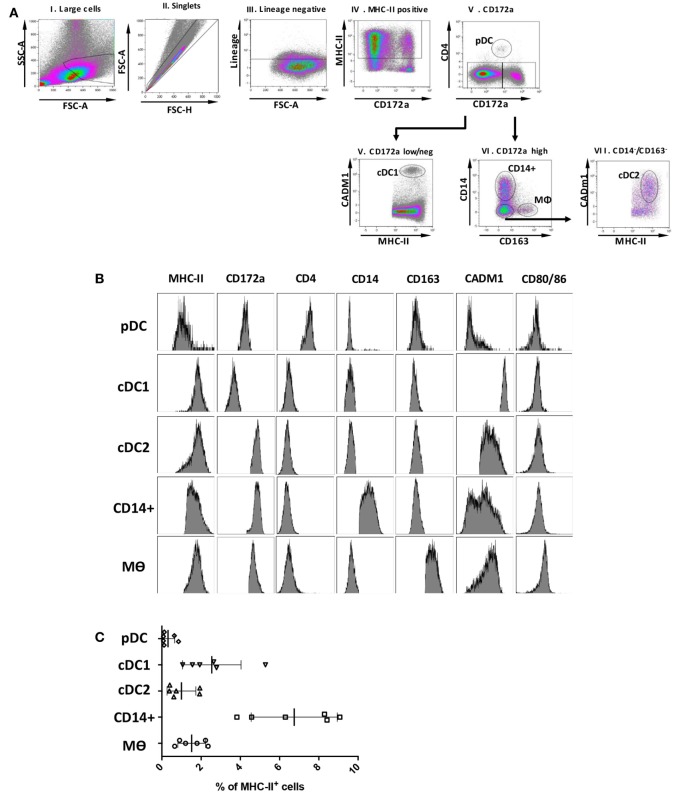
Phenotype of porcine tonsillar myeloid cells. **(A)** Dissociated tonsillar cells were depleted of cells expressing specific lineage markers (CD3, CD21, CD8α, and IgM), and the subsequent enriched myeloid cells were stained with mAbs and examined by flow cytometry. Illustrative density plots show the gating strategy: I. Large cells, II. Singlets, III. Lineage negative, IV. MHC class II, V. CD172a. Five myeloid cell populations were identified as shown by the annotated gates, pDCs gated as, MHC-II^low^ CD172a^low/neg^ CD4^+^ CADM1^−^ CD14^−^ CD163^−^, a cDC1-like population gated as MHC-II^high^ CD172a^low/neg^CD4^−^ CADM1^high^ CD14^−^ CD163^−^, a putative cDC2-like gated as MHC-II^high^, CD172a^high^, CADM1^low^, a putative moDC CD14^+^ gated as, CD172a^high^, CD163^−^, CD14^+^, and putative Mϴs as, CD172a^high^, MHC II^+^, CD163^+^. **(B)** Flow cytometry histograms showing MHC class II, CD172a, CD4, CD14, CD163, CADM1, CD80/86 expression associated with each of the five defined myeloid cell populations. The histograms shown are illustrative for a single pig and are representative of six animals. **(C)** Plot demonstrating the relative proportion of each of the defined populations within the MHC II positive gate for six different pigs. Bars indicate the mean and error bars represent the SD for each individual population.

A panel of markers that had previously defined DCs and Mϴ populations in various species (7, 28, 29) was selected to determine the presence of myeloid populations resident in porcine tonsil (Figure 1). Since myeloid cells are comparatively rare in secondary lymphoid tissues, they were enriched by antibody-associated magnetic depletion of cells expressing the lymphocyte lineage markers CD3 (T cells), CD8α (NK cells), CD21, and sIgM (B cells). Following doublet discrimination (Figure 1A), live cells were identified and remaining lineage positive cells excluded. After selection of MHC class II-positive cells, a candidate pDC population was identified as CD172a^low^CD4^+^ MHC II^low^, which corresponds to the defined porcine pDCs (30) (Figure 1B). The remaining cells delineated into CD172a^neg/low^, CADM1^high^, MHC II^high^ cells representing a putative cDC1 population. The CD172a^high^ cells could be divided into three distinct populations whereby CD172a^high^, CD14^−^ CD163^−^ MHC II^high^, CADM1^low^ phenotypically resembled a cDC2-like population (cDC2); CD172a^high^, CD163^−^CD14^+^ MHC II^high^ cells were believed to be a monocyte-derived population (CD14^+^ cells), while CD172a^high^, CD163^+^CD14^−^ and MHC II^high^ cells represented a Mϴ–like population (Mϴs).

To assess the relative frequency of each of the five cell populations in the tonsil, the percentage of each subset was determined within the MHC-II positive fraction (Figure 1C). The CD14^+^ cells were the most frequent DC population at 6.75 ± 2.20%, followed by cDC1s (2.54 ± 1.49%), Mϴs (1.52 ± 0.71%), cDC2s at 1.00 ± 0.74%, and pDCs being the rarest population (0.32 ± 0.34%).

### Expression of Conserved Myeloid Cell Markers Assessed by qPCR

**Figure 2:**
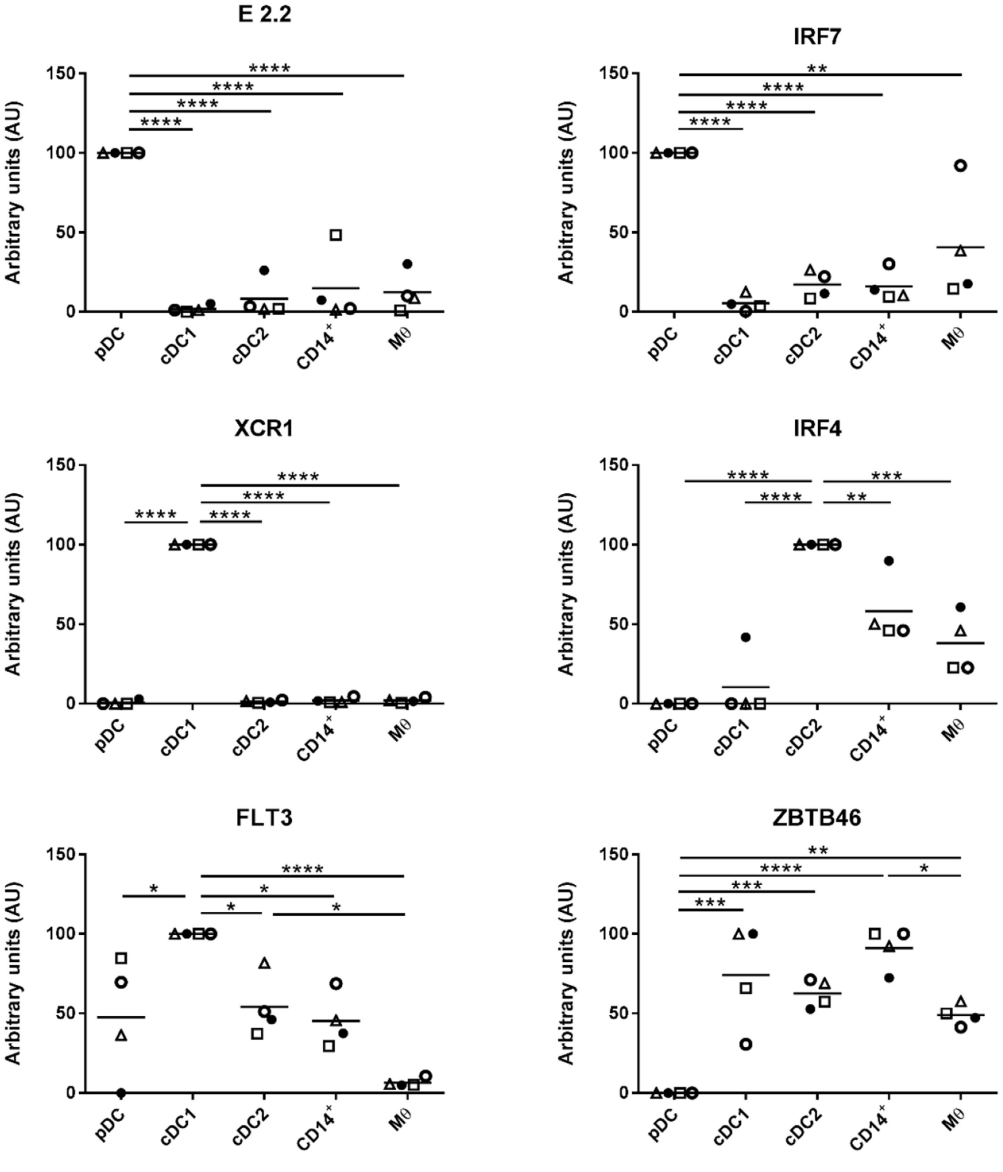

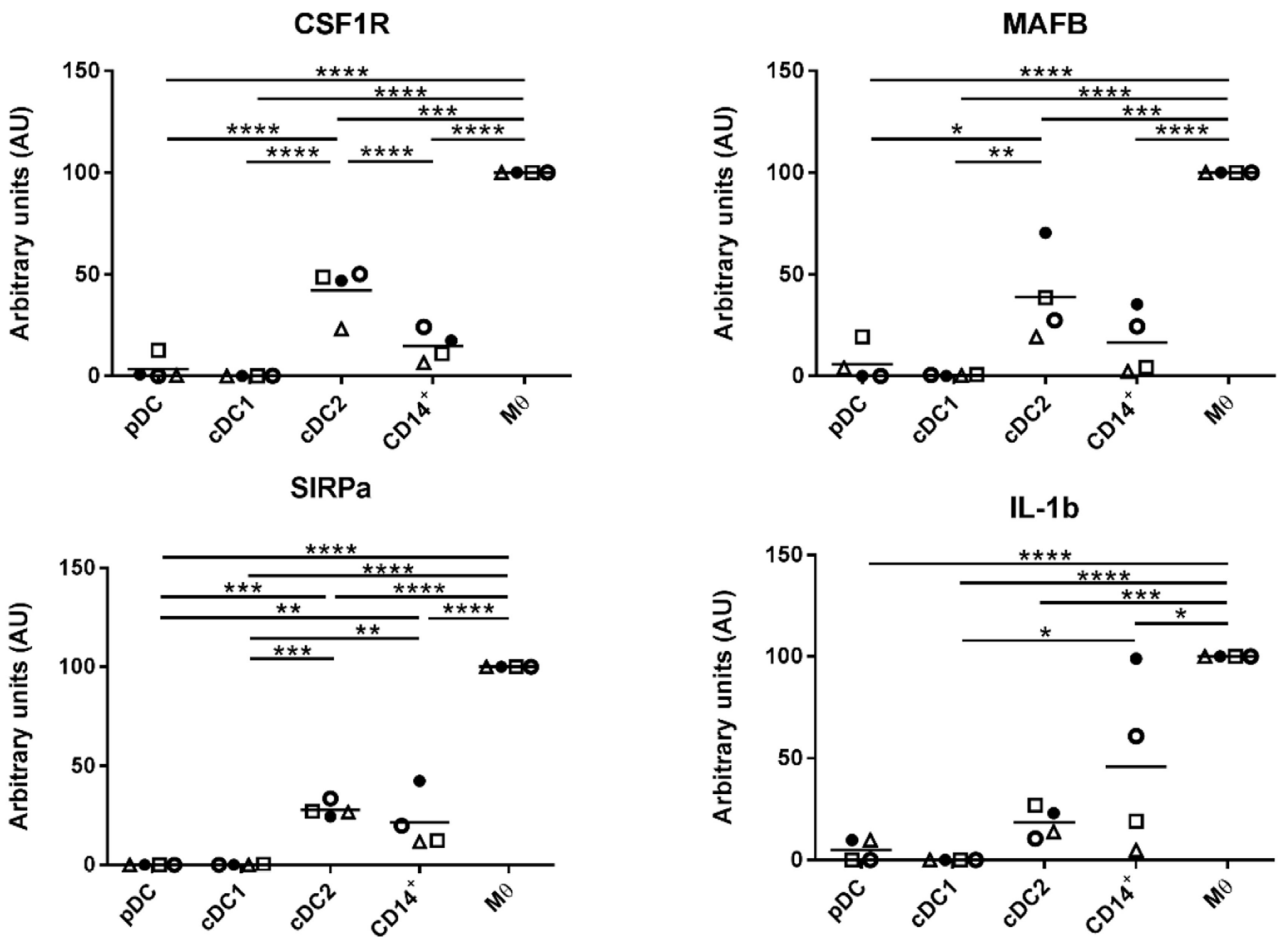
Expression of DC/Mϴ/monocyte related genes by tonsil myeloid cell populations. The five identified populations were sorted, mRNA extracted, and the expression of several DC/Mϴ associated gene transcripts by the different populations assessed. mRNA expression levels of E2.2, IRF7, XCR1, IRF4, FLT3, ZBTB46, CSF1R, MAFB, SIRPa, and IL-1b were evaluated by quantitative real-time RT-PCR. Gene expression was normalized to BACT (β-actin) and the relative expression of each gene was calculated with the 2^−ΔCt^ formula using the mean C_t_ values from duplicate samples. For each pig, data are expressed in arbitrary units (AU) obtained by assigning a value of 100 to the population giving the maximum level of expression and the remaining populations were compared to it for each gene. Each point on the graph represents the normalized 2^−ΔCt^ value from each cell population from each individual animal. This experiment was performed on a minimum of three animals in more than three independent experiments. Statistical analysis was performed by a one-way ANOVA and statistical significance is defined by ****p < 0.0001, ***p < 0.001, **p < 0.01, and *p < 0.05.

A key aim of this study was to align the identified myeloid cell populations with their human and mice counterparts and to correlate them with similar populations in porcine skin (7, 8), lungs (24) blood (21, 22), and lymphoid tissue, including tonsil (25). However, not all proteins can be assessed by flow cytometry in pigs, due to lack of suitable antibodies. Several putative markers were, therefore, selected for further evaluation by gene expression. Expression of these markers is conserved across DC subsets and/or Mϴ populations in different species (24) and their appearance on porcine tonsil myeloid cells was assessed by RT-qPCR. The putative pDC population was found to express high levels of E2.2, a gene important in pDC development (21), and also IRF7 and FLT3 (Figure 2). The cDC1-like cells expressed the cDC marker ZBTB46 (31) and the highest levels of the bona fide DC marker FLT3. These cells also demonstrated expression of XCR1, a gene considered to be a hallmark of cDC1 (32). The cDC2-like cells expressed FLT3 and ZBTB46, and high levels of IRF4 and SIRPa (SIRPα), genes involved in cDC2 development. Monocyte/Mϴ related genes including IL-1b (IL-1B), MAFB, and CSF1R were also expressed by the cDC2-like population, albeit at lower levels than observed in Mϴs. Notably, the CD14^+^ cells expressed DC related genes FLT3 and ZBTB46, but also Mϴ related genes IL-1B, MAFB and CSF1R. IRF4 expression was also observed although at lower levels than associated with the cDC2-like cells. Finally, as expected, Mϴs failed to express FLT3 but did express ZBTB46 but at slightly lower levels than the cDC and CD14^+^ cells. Unsurprisingly, the putative Mϴ population expressed the highest levels of IL-1B, MAFB, and CSF1R and also the highest levels of SIRPα.

### MHC-II and Costimulatory Molecule Expression on Isolated and Cultured Tonsillar Myeloid Cells

**Figure 3:**
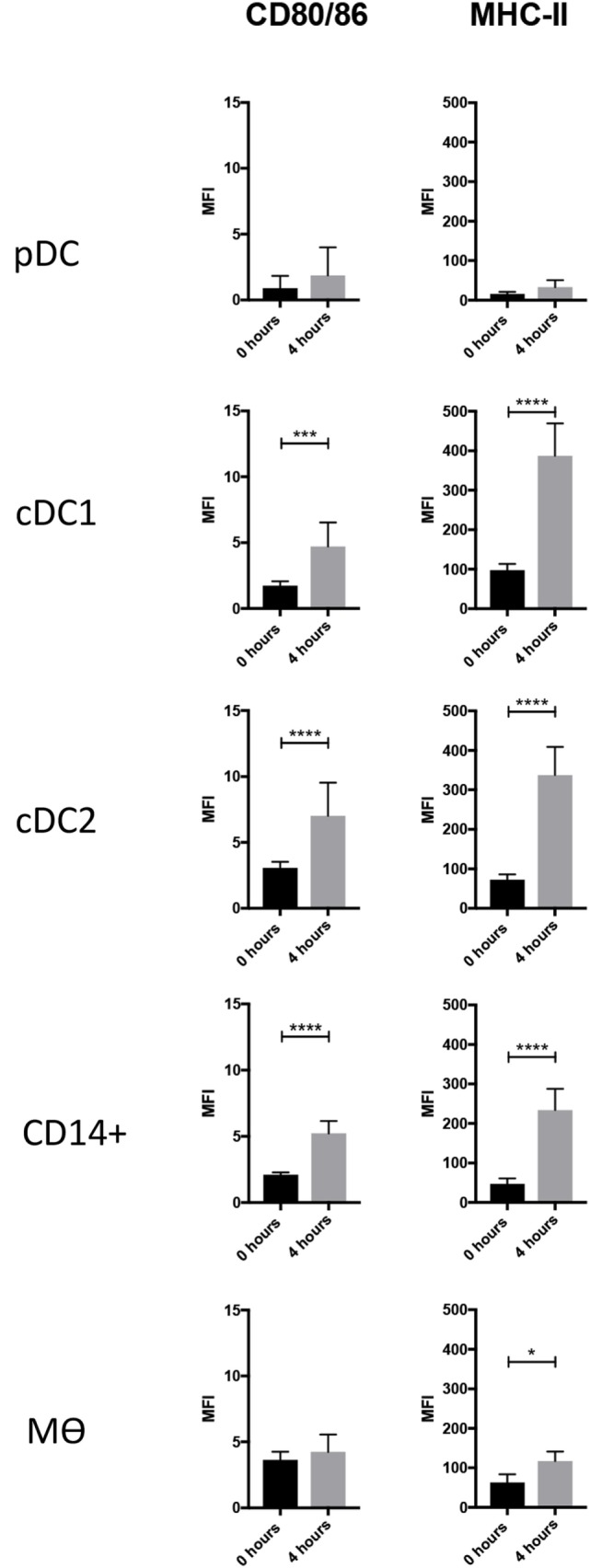
Costimulatory molecule expression following 4-h culture. The enriched tonsillar myeloid cells were stained, before and after culture, with the same antibody panel and gated as described above. Cells were also stained for with CTLA4-Fc fusion protein to assess CD80/86 expression. Bar graphs showing the MFI values for CD80/86 and MHC class II corresponding to each of the defined myeloid populations. Values shown are means from eight individual pigs and error bars represent 1 SD. Values were compared using a two-way ANOVA and significance indicated by ****p < 0.0001, ***p < 0.001, *p < 0.05.

DCs are characterized by an ability to activate naïve T cells due to their constitutive expression of MHC class II and costimulatory proteins (33). At steady state, and immediately after isolation, the highest levels of MHC class II were associated with cDC1s, closely followed by cDC2. The lowest levels of MHC class II were associated with pDCs (Figure 3). The highest levels of CD80/86 expression were associated with the Mϴ-like population while again the lowest levels were expressed by pDC. To evaluate whether these markers might increase in expression as the cells develop a more mature phenotype, cells were cultured for 4 h in the absence of specific stimulation. For both pDCs and Mϴs, there was only a modest increase in CD80/86 and MHC-II expression levels as demonstrated by the MFI (mean fluorescence intensity), which was not statistically significant. In contrast, CD14^+^ cells, cDC1-like and cDC2-like cells, all demonstrated a significant upregulation of both these markers. CD14^+^ cells increased expression levels of CD80/86 from 2.111 ± 0.172 to 5.232 ± 0.941, and MHC-II expression from 47.141 ± 13.775 to 234.164 ± 53.693. cDC1-like cells increased their CD80/86 expression from 1.742 ± 0.328 to 4.715 ± 1.821 and MHC-II from 97.517 ± 15.997 to 387.157 ± 82.053 and finally cDC2-like cells increased CD80/86 from 3.065 ± 0.460 to 7.013 ± 2.514 and MHC-II from 72.528 ± 13.334 to 337.820 ± 71.097 (Figure 3).

### Evaluation of TLR Expression Across the Five Populations of Myeloid Cells

**Figure 4:**
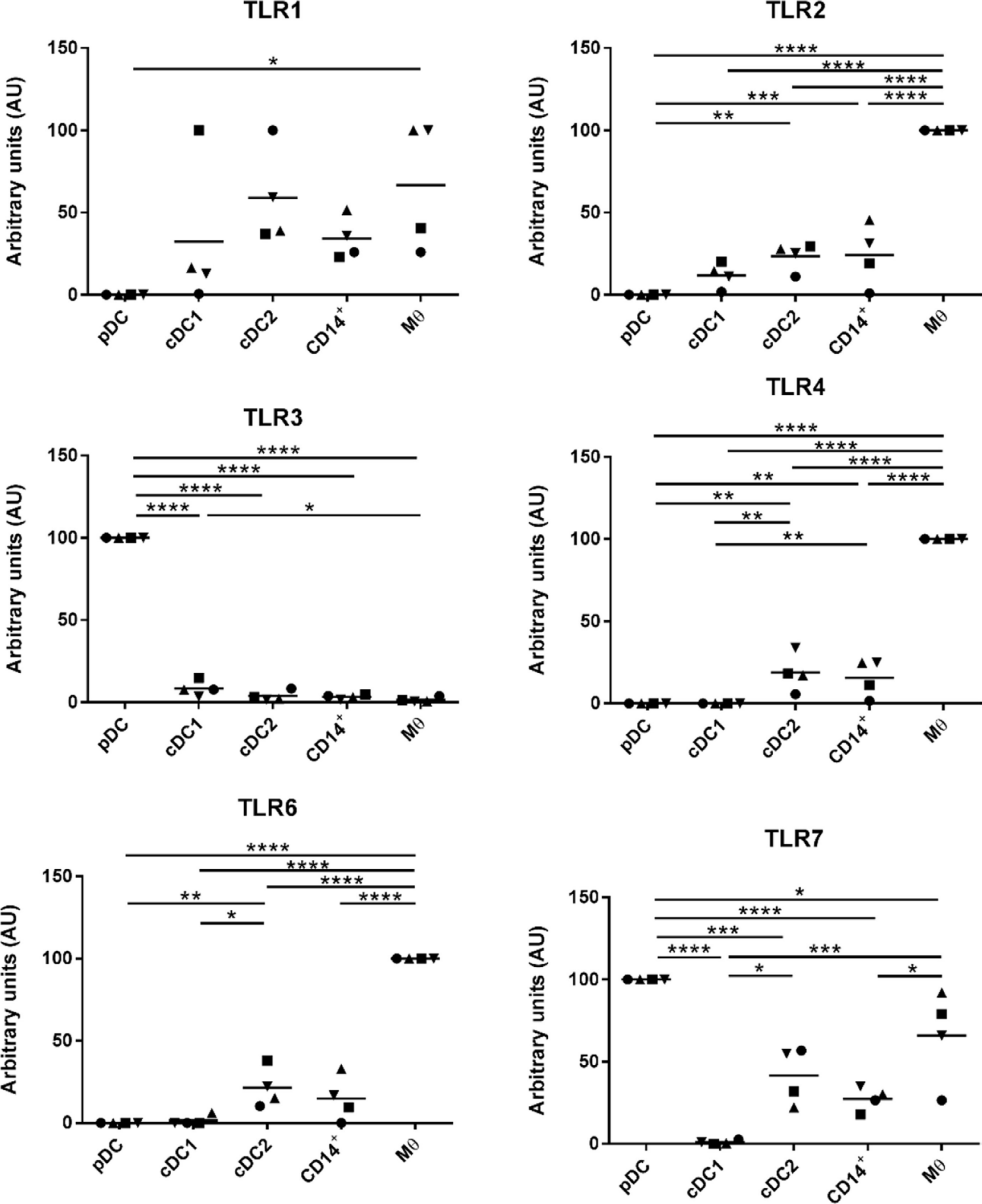

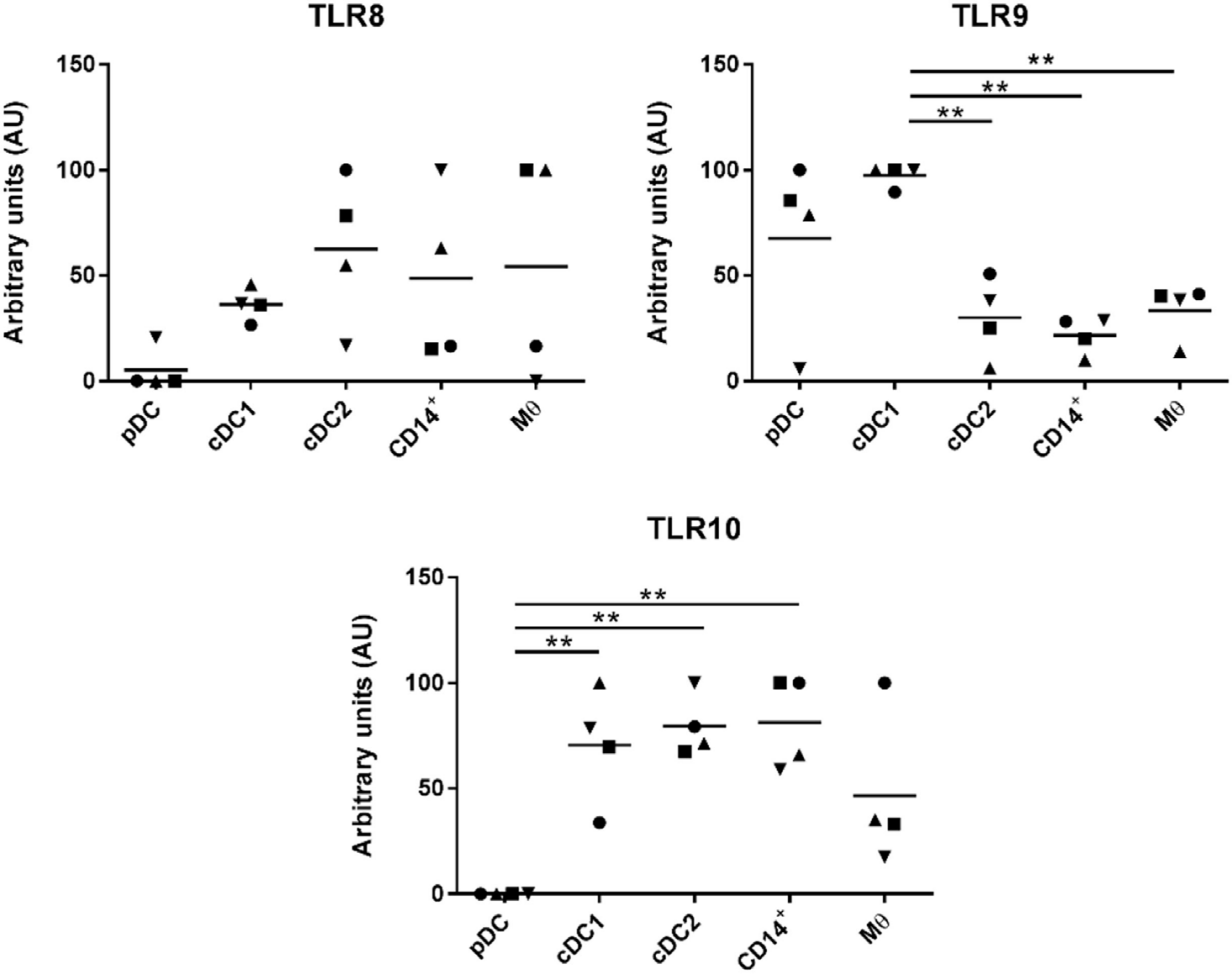
Evaluation of toll-like receptors (TLRs) expression by RT-qPCR. The five populations were sorted and the mRNA was extracted to evaluate the expression of TLRs 1 to 10 by quantitative real-time PCR. Each TLR gene expression level was normalized to β-actin and the relative expression of each gene was calculated with the 2^−ΔCt^ formula using the mean C_t_ values from duplicate samples. For each pig, data are expressed in arbitrary units obtained by assigning a value of 100 to the population giving the maximum level of expression and the remaining populations were compared to it for each gene. Each point on the graph represents the normalized 2^−ΔCt^ value from each individual animal. This experiment was performed on 3 or 4 animals in three independent experiments. Statistical analysis was performed by a one-way ANOVA and statistical significance is shown by ****p < 0.0001, ***p < 0.001, **p < 0.01, and *p < 0.05.

Myeloid cells express a broad repertoire of pathogen recognition receptors including TLRs. Binding of the TLRs serves as a danger signal resulting in myeloid cell activation and ultimately a trigger for activation of the adaptive immune system. Others have demonstrated that DCs and Mϴ populations express conserved TLR profiles; for example, human and mouse Mϴs are associated with high levels of TLR4 expression (34) while cDC1 cells express TLR3 (35). To further evaluate the phenotypes of the five myeloid cell populations the TLR profile (TLR1-10) of each of the populations was determined by RT-qPCR (Figure 4). For all cell populations, TLR5 expression was below the limit of detection and, therefore, the data are not shown. TLR1, 8, and 10 were expressed at comparatively similar levels across all cell populations with the exception of pDCs, which expressed much lower levels. TLR2, 4, and 6 were expressed at significantly higher levels on the Mϴ-like cells compared to all other populations. TLR2, 4, and 6 were also expressed, albeit at a lower level, on the CD14^+^ cells and the cDC2-like cells, while negligible levels of expression were seen on cDC1-like and pDCs. Similarly, to human and mouse pDCs, TLR7 and 9 were expressed at high levels on porcine tonsil pDC populations. However, TLR7 was not restricted to pDCs, but was also expressed by Mϴ-like cells, cDC2-like cells and CD14^+^ cells (again at a lower level). This is consistent with a previous report demonstrating expression of TLR7 by porcine blood monocytes and cDC2s, in addition to pDCs (21). Another difference to human and mouse cells was the very high level of expression of TLR3 on pDCs, which is otherwise restricted to cDC1 cells, yet in pigs was expressed at very low levels on tonsillar cDC1s. Notably, cDC1s expressed TLR9 at comparable levels to pDCs.

### Evaluation of Antigen Processing, T Cell Stimulatory Capacity, and Cytokine Responses to TLR Stimulation

**Figure 5:**
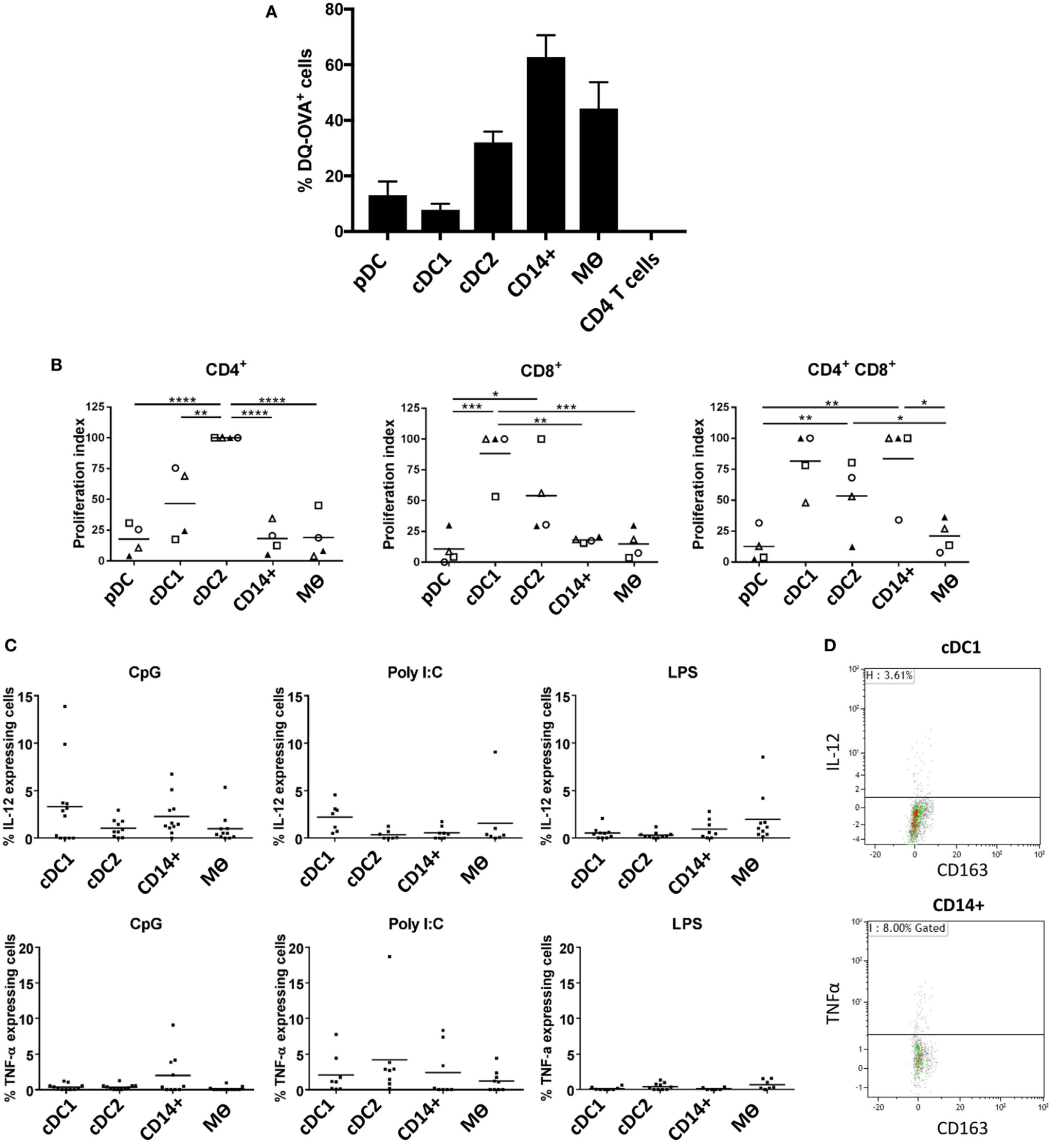
Evaluation of antigen processing, T cell stimulatory capacity and cytokine secretion profile following TLR stimulation **(A)**. Differential endocytosis between cell populations was evaluated with OVA-DQ-FITC by culture of lineage depleted myeloid cells for 1.5 h at 37°C (and 4°C), and FITC fluorescence was assessed by flow cytometry. Histogram shows the mean percentage of cells taking up DQ-OVA from eight pigs (each tested in triplicate), following subtraction of non-specific fluorescence (uptake at 4°C) for each cell population from three independent experiments. CD4 T cells were also assessed as a negative control. **(B)** Myeloid cells (APC) were sorted and peripheral blood mononuclear cells from allogeneic animals were stained with Violet CellTrace and mixed at a APC:T cell ratio of 1:10 before being culture for 5 days at 37°C. Proliferation of CD4^+^CD8α^−^ (CD4 T cells), CD4^−^CD8α^+^ (CD8 T cells), and CD4^+^CD8α^+^ (memory T cells) was evaluated by flow cytometry. A value of 100 was assigned to the population with the highest proliferation value and all other populations were compared to this value (and repeated for each pig). Data are from three separate experiments and a minimum of four different animals for each cell type. A one-way ANOVA was performed and statistical significance is described by ****p < 0.0001, ***p = 0.0002, **p = 0.0016, and *p = 0.0108. **(C)** Isolated tonsil cells were depleted for lineage markers (CD3, CD8α, CD21, and IgM) and stimulated for 12 h in the presence of toll-like receptors agonists CpG, Poly I:C or LPS. After incubation, the myeloid populations were defined using the same antibody panel as described above. IL-12 (top panel) and TNF-α (bottom panel) secretion was assessed by intracellular staining and flow cytometry. For each cell population, each point represents a single pig and the horizontal line represents the mean of at least seven pigs tested in three independent experiments. The mean percentage of secreting cells (non-stimulated) was subtracted from each of the relevant data points. **(D)** Representative flow cytometry dot plots, showing IL-12 and TNFα secretion associated with cDC1 and CD14^+^ cells, respectively following CpG stimulation.

One of the cardinal functions of myeloid cells is their ability to process and present antigen in order to activate T cells. To evaluate the antigen processing capacity of each of the five identified populations, we assessed the uptake and processing of quenched DQ-OVA-FITC particles following 1.5 h of culture (Figure 5A). Both pDCs and cDC1-like cells were the least efficient at processing DQ-OVA-FITC particles followed by the cDC2-like population. In contrast the CD14^+^ cells were the most efficient followed by the Mϴs. Next, to assess the relative ability of the myeloid cells to activate T cells, we compared the capacity of the different populations to activate allogeneic CD4^+^ and CD8^+^ T cells in mixed lymphocyte reaction (MLR). The cDC1-like and cDC2-like populations were most able to stimulate naïve CD4^+^ T cells with the DC2 cells showing the highest stimulatory capacity (Figure 5B). The cDC1 and cDC2-like populations were most effective at stimulating CD8^+^ T cell proliferation; however, while cDC1-like cells showed a tendency toward the higher proliferation index, this was not found to be statistically significant. Finally, the cDC1-like cells, cDC2-like cells, and the CD14^+^ cells were equally able to stimulate memory CD4^+^ T cells (shown by others to express a CD4/CD8 double-positive phenotype) (36), while the pDCs and Mϴs showed a relatively low capacity for stimulating allogeneic T cells. Also, since the PBMCs were stained with antibodies to CD4 and CD8 (and did not include CD3), we cannot discount the possibility that NK cells will also be included within the CD8 T cell population.

Finally, we evaluated how the myeloid cells might respond to TLR stimulation. Cells enriched in myeloid subsets (through depletion of cells expressing lineage markers) were cultured with CpG (ODN21798), which is a TLR9 agonist, Poly I:C as a TLR3 agonist and LPS as a TLR4 agonist and assessed for expression of IL-12 and TNF-α by flow cytometry. Due to decreased cell viability following 10 h culture, pDCs had to be excluded from these analyses. The results showed that cDC1s secreted IL-12 following CpG stimulation (Figures 5C,D), most likely reflecting TLR9 expression on this cell population (Figure 4). However, CD14^+^ cells also responded to CpG stimulation secreting both IL-12 and TNF-α despite their comparatively low levels of TLR9 expression. Similarly, cDC1-like cells secreted the highest levels of IL-12 following Poly I:C stimulation despite an apparent low abundancy of TLR3 associated with these cells.

### *In Situ* Localization of Myeloid Cell Populations in Tonsils

**Figure 6:**
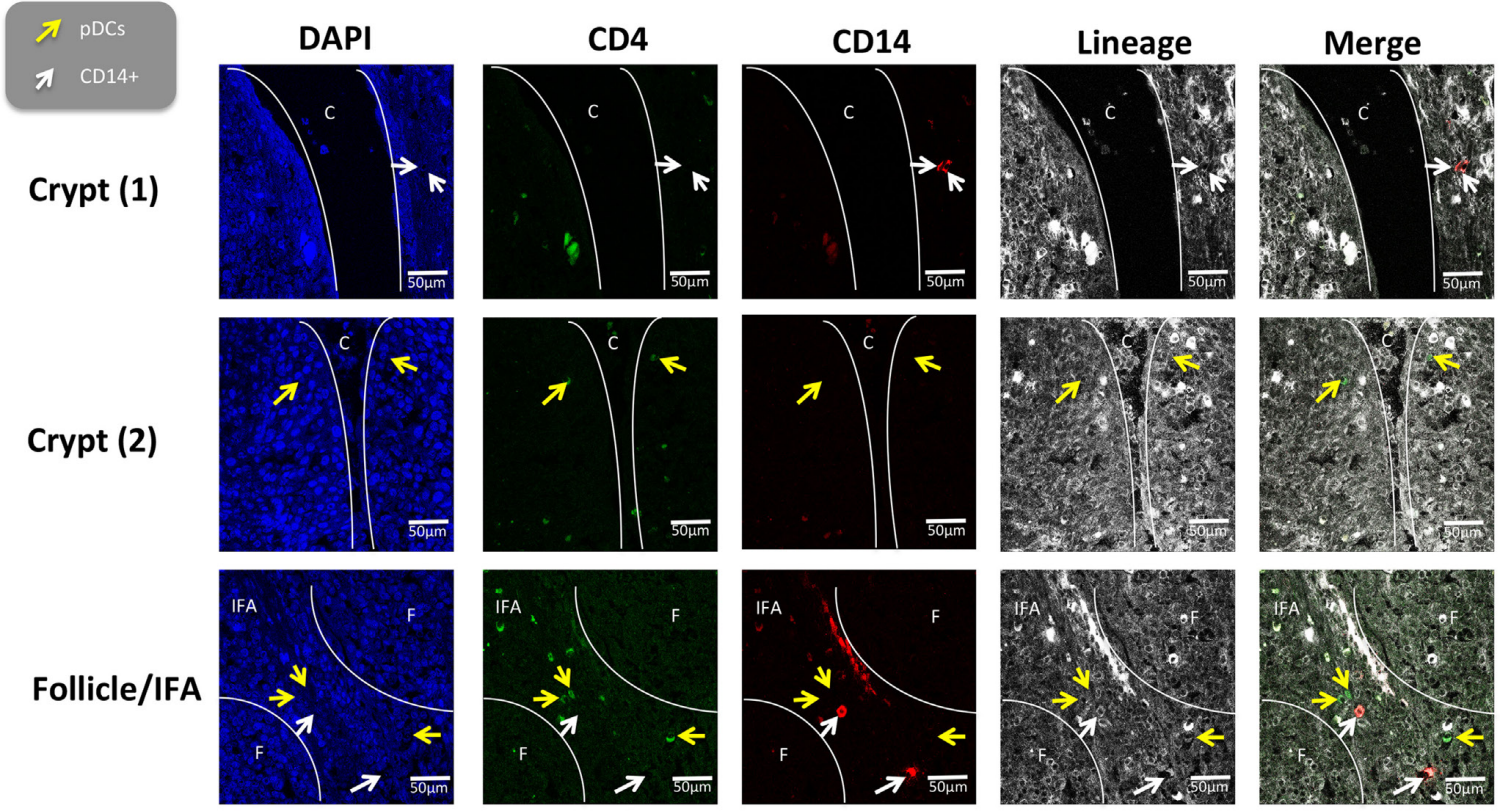
*In situ* localization of the CD14^+^ cells and plasmocytoid dendritic cells in porcine palatine tonsil. CD14^+^ cells and pDCs were localized by confocal microscopy following ethanol fixation of tonsil slices. The areas assessed included the follicle (F), the interfollicular region (IFA), the crypt (C). The tissue was stained using panel 1 antibodies; white arrow CD14^+^ cells and yellow arrow pDCs. Images are representative of at least two images from each section, from three different pigs. Objective used: (A) 63× oil immersion. Scale bars as shown.

**Figure 7:**
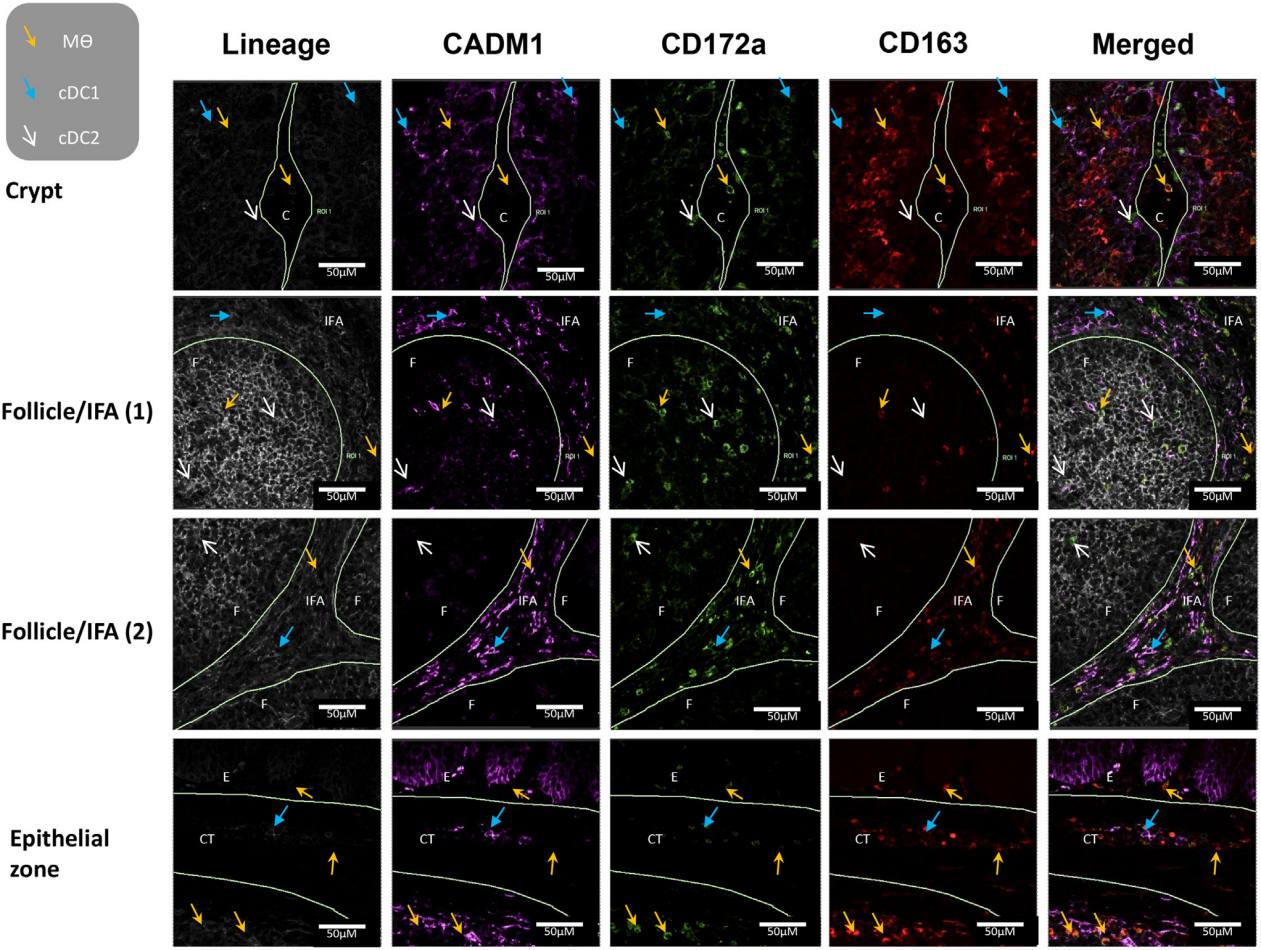
*In situ* localization of conventional dendritic cells and macrophages in porcine palatine tonsil. Two cDC subsets and Mϴs were localized by confocal microscopy following ethanol fixation of tonsil slices. The areas assessed included the follicle (F), the interfollicular region (IFA), the crypt (C), the connective tissue (CT), and the epithelium (E). Tissue stained using panel 2 antibodies; blue arrow cDC1, white arrow cDC2, yellow arrow Mϴs. Images are representative of at least two images from each section, from three different pigs. Objective used: 40× oil immersion. Scale bars as shown.

To further evaluate the five myeloid cell populations identified in the tonsil, we investigated their sub-localization *in situ*. To minimize spectral overlap between the fluorophores, the five myeloid cell populations were identified across two separate panels; panel 1 to identify CD14^+^ cells and pDCs and panel 2 to identify cDC1s, cDC2s, and Mϴs (Figures 6 and 7).

The tonsil regions assessed included the tonsillar crypts (C), lymphoid follicles (F), the interfollicular area (IFA), and the epithelia (E). Using panel 1, pDCs were detected in the IFA and less frequently in the follicles (data not shown) and beneath the squamous epithelia lining the crypt (Figure 6). Similarly, CD14^+^ cells were located mostly in the IFA and beneath the epithelia of the crypt. On occasion, these cells were detected in the follicle. Neither of these populations could be detected in the epithelium or connective tissue (CT).

Applying panel 2 (Figure 7), cDC1s were observed in the area surrounding the crypt, the follicles, the CT, and also the IFA. cDC2s were restricted to the crypt epithelium and the follicles. Finally, Mϴs were found in the crypt lumen leading to the outside of the tissue and also in the area surrounding the crypt. They were particularly abundant on the edge of the tonsils, where they could be detected in the epithelium, the subepithelial CT, sinoid, and the adjacent lymphoid tissue. Clearly, Mϴs and CD14^+^ cells were positioned close to areas were pathogens might be expected to enter the tonsil.

## Discussion

Understanding the complexity of myeloid cell populations in pigs has become an important topic both in furthering our understanding on how these cells coordinate the adaptive immune system but also with respect to the opportunities that these cells offer as targets to modulate the immune response, e.g., in vaccine development. Specialized subsets of DCs and monocyte/Mϴs have now been comprehensively studied in various species and tissues and the evolutionary conserved patterns of cell phenotype and function established between human and rodents have generally remained true across livestock and companion animal species alike (7, 28, 29, 37).

Porcine myeloid cell populations have recently been characterized in blood (21, 22), lung (24), and skin (7, 8); however, little is known regarding their frequency and phenotype in secondary lymphoid organs, including the tonsil. A very recent study reported the presence of two resident dendritic cell populations; cDC1s and cDC2s in swine palatine tonsil (25), however, to our knowledge, this is the first study to apply multi-color flow cytometry, confocal microscopy in addition to molecular and functional assays to delineate five distinct populations of myeloid cells resident in porcine tonsil.

The work presented here demonstrates clear homologies of porcine tonsillar myeloid cells with myeloid populations described in other porcine tissues and human tonsil. The myeloid cells were immature in the steady state, a feature consistent with the orthologous population in human tonsil (26). Porcine pDCs were identified as MHC-II^low^ CD172a^low/neg^ CD4^+^ CADM1^−^ CD14^−^ CD163^−^ as previously described in porcine blood (21, 22). True to their assigned lineage, PCR analysis confirmed expression of FLT3, a tyrosine kinase receptor [necessary for development of DCs from progenitor cells (38)] and E2-2, a specific transcription regulator of pDCs development in mouse, human and pigs (21, 39). pDCs also expressed IRF7 and TLRs 7 and 9 as observed in human pDCs (40) and demonstrated a low antigen processing and T cells stimulatory capacity as shown in other porcine tissue (22, 30, 41). The cDC1-like cell population was identified by the high expression of MHC-II, the low/neg expression of CD172a, negative expression of CD4, CD14, and CD163 and high expression of CADM1 as described in porcine skin (8), lung (24), and blood (21, 22). This phenotype was also described by Parra-Sanchez et al. (25) in porcine tonsils. High levels of FLT3 and XCR1 mRNA expression confirmed the definition of this subset and orthology across species (35, 42). Secretion of IL-12 and a propensity to drive T_H_1 responses have also been linked to this population (14), and here, high levels of IL-12 were associated with cDC1s following CpG stimulation, also demonstrated in cDC1s from porcine lung (24). It was interesting that high levels of IL-12 was also produced by cDC1s in response to Poly I:C despite the low abundancy of TLR3. Furthermore, all tonsillar populations responded to TLR3 agonist with TNF-α. Clearly, the interaction of DCs, with each other (and possibly any remaining lymphoid cells in the population) is influencing the cytokine secretion profile in addition to TLR expression. The assessment of sorted cell subsets may have revealed the genuine cytokine expression profile for each population, although evaluation of a mixed population of cells permits a more realistic approach for assessing cytokine secretion patterns *in vivo*.

The phenotype of the cDC2 lineage was confirmed as MHC-II^high^CD172a^high^CD4^−^ CADM1^low^CD14^−^CD163^−^ with a moderate ability to take up and process antigen but a superior capacity to activate allogeneic naïve CD4 T cells. Activation of CD4 T cells, moderate CADM1 expression, and induction of T_H_2 responses are hallmarks of this cell population in porcine lung (24) and blood (21, 22). PCR analysis of this sorted cell subset revealed FLT3 and ZBTB46 expression and the highest levels of the transcription factor IRF4, necessary for development of cDC2s from CD11c progenitor cells in lung and spleen in the mouse (43) and for promoting CD4^+^ T cell responses in humans (44). Finally, TLR expression by these cells was consistent with what has been observed in porcine blood cDC2s (21) suggesting a conserved TLR expression profile in this population across several tissues.

The lineage of the fourth, most frequent CD14^+^ cell subset identified in the tonsil (MHC-II^high^CD172a^high^CD4^−^CADM1^low^CD14^+^CD163^−^) was less clear. Expression of FLT3 and ZBTB46 would classify them as cDCs rather than pDCs, monocytes, and Mϴs (31), yet, the low expression of MAFB (45) and CSF1R (46) and variable expression of IL-1B (47) favors a myelo-monocytic cell lineage. Notably, a population of CD163^+^ dermal DCs in porcine skin, which transcriptomically aligned to moDCs in human and mouse (8) expressed CSF1R, MAFB, and ZBTB46 suggesting that this fourth population may also align with this subset. Furthermore, expression of CD14 (48), FLT3 (49), TNF-α secretion (18), and a role in pathogen clearance (high uptake of DQ-OVA) (50) are consistent with an inflammatory DC lineage generated from circulating myelo-monocyctic cells as shown by others (51). However, we cannot discount the possibility that these cells might also contain cDC2s, given that different levels of CADM1 were expressed on these cells and that CD14 has also been associated with human blood cDC2s (35, 52). However, CD14 remains a marker used to indicate a likely monocytic origin (52) and is yet to be demonstrated as a specific marker for cDC2s in pigs (21, 22, 24, 30). It is also plausible that the two levels of CADM1 (demonstrated on these cells) represent two populations of moDCs, which have yet to be fully delineated and, therefore, assumed to be a single DC subset (53). Notably, the dominance of such a cell population in healthy pigs at slaughter seems counter-intuitive, but while these pigs are clinically healthy, they are not SPF at slaughter age and as such are subject to challenge by both environmental stimuli and pathogens (54). Furthermore, the variety of husbandry practices adopted between farms might explain the variability in frequency of this population between animals.

Finally, we detected a population of tonsillar cells expressing MHC-II^high^CD172a^high^CD4^−^CADM1^low^CD14^−^CD163^+^, characterized by a high capacity to capture and process antigen, a low capacity for naïve T cell stimulation and an absence of FLT3 expression suggesting these cells to be Mϴs. This is further supported by the high relative abundancy of CSF1R, MAFB, SIRPα, and IL-1B transcripts, which is consistent with a Mϴ lineage. Notably, the highest level of SIRPα (CD172a) transcripts were associated with Mϴs, which is in contrast to the flow cytometry data, which demonstrated similar levels of SIPRα surface expression across Mϴs, cDC2s, and CD14*^+^* cells. The reason for this inconsistency is unclear but might relate to the higher detection sensitivity of RT-qPCR, or that surface expressed SIRPα expression changes in response to various immune mechanisms following cell activation (55). This subset also showed the highest level of expression of TLR2, TLR4, and TLR6 (56), again consistent with a Mϴ identity. Interestingly, this was the only cell population, which appeared to be present in the crypt, the CT, and the epithelium and is, therefore, likely to play a significant role in the uptake of antigens and host defense. Notably, all of the myeloid cell populations were observed in the area beneath the crypt epithelium indicating that all these cells are well positioned to assist in the uptake of antigens, which have translocated the crypt epithelium, for subsequent T lymphocyte activation. This is consistent with previous reports (57).

Despite the clear alignment of the myeloid populations with their human and mouse counterparts, differences were observed. For example, pDCs expressed TLR3, which is otherwise restricted to the cDC1 cell subsets in mice (58) and humans (14), although this has also been reported in porcine blood pDCs (21) and might imply a porcine-specific pDC response to a wider set of pathogens. Furthermore, we demonstrated that cDC1 and cDC2 subsets share a similar capacity to activate allogeneic CD8 T cells, which is consistent with cDC populations in lung (24) while others have shown that in porcine blood (22) and lymph DCs in sheep (29), cDC1s are superior at activating CD8 T cells. Perhaps in tonsil and lung, being two of the main portals of pathogen entry, a shared ability between cDCs populations to stimulate CD8 T cells may be advantageous. We also report that CD14^+^ cells were the most frequent population in the tonsil, closely followed by cDC1s, which is in contrast to other tissues, where cDC2s are typically found to be more widespread than cDC1s (24, 59). A higher frequency of cDC1 (compared to cDC2) was also reported in porcine lymphoid tissue (25). The reason for this altered balance of DC subsets remains unclear but could reflect a local presence of DNA-associated pathogen and thus a requirement for TLR9 expression, a receptor, which appears to be specific to cDC1s. Alternatively, this disparity might just reflect inherent differences between different tissues. Our results demonstrate that cDC1s populations secrete IL-12 as also shown in the porcine lung (24) while others have shown that in the blood, pDCs secrete the highest levels of IL-12 (21, 22). Due to the scarcity and limited survival of pDC cells outside the tonsil, we were unable to include these cells in our analyses and, therefore, can neither refute nor confirm this for porcine tonsil. Finally, the CD14^+^ cells population in the porcine tonsil was found to be CD163^neg/low^ with levels of FLT3 mRNA comparable to cDC2s. This is in contrast to CD163^low^ cells (believed to be moDCs in the lung), which were negative for FLT3 (24). CD163^low^ cells in skin were also shown to be negative for FLT3 although expression was still 100 times higher than observed in Mϴs (8). The reasons for this difference are currently unclear and require further investigation.

This study has demonstrated a distinction and specialization between myeloid cell populations as shown previously by others. However, there is also clearly a degree of plasticity in both cell phenotype and function. For example, CADM1 is expressed on cDC2s, albeit at levels significantly lower, than associated with cDC1s. We also reported co-expression of TLR2 4 and 6 across CD14^+^ cells, cDC2s and Mϴ populations indicating that all three cell subsets are able to recognize and respond to similar invading pathogens. Clearly as this area of work develops and techniques to delineate myeloid cell populations become more sophisticated, the association between these identified myeloid cells may become more apparent and additional cell populations may also emerge. It is likely that both the anatomical and pro-inflammatory environment will add a further layer of complexity both to the classification of these cells and their ontogeny. How this added “flexibility” might then influence DC responses to pathogens entering the tonsil remains to be explained.

In summary, this study dissected the myeloid cells present in the porcine palatine tonsil and identified five distinct populations of the myelo-monocytic and DC lineages; two subsets of conventional DCs (cDC1s and cDC2s), pDCs, as well as a putative moDC population and Mϴs, with clear homology to human subsets. As such, the interaction of tonsillar myeloid cells with viruses such as classical swine fever, which share many characteristics with human viral hemorrhagic fever (1), might assist in furthering our understanding of host–pathogen interactions.

## Ethics Statement

Tonsil tissue was supplied by a local abattoir. Blood sampling was approved by the Animal and Plant Health Agency’s Animal Welfare and Ethics Review Board and all procedures were conducted in accordance with the UK Animals (Scientific Procedures) Act 1986 under Project Licence PPL 70/8343.

## Author Contributions

FSo, BC, and LS contributed to the performance of the experiments. FSo also contributed to the design of the experiments, performed the data analysis and preparation of the manuscript. JE contributed to the design of the experiments, performed the cell sorts, and assisted with the preparation of the manuscript. SG, HC, DW, and FSt contributed to the design of the experiments and preparation of the manuscript. All authors reviewed the manuscript.

## Conflict of Interest Statement

The authors declare that the research was conducted in the absence of any commercial or financial relationships that could be construed as a potential conflict of interest.
